# Quantitative analysis and identification of magnesium alloys using fs-LA-SIBS combined with machine learning methods

**DOI:** 10.1039/d4ra07007k

**Published:** 2025-01-16

**Authors:** Jun Liu, Ji Wang, Xiaopei Li, Hai Lin, Tiancheng Liu, Bingyan Zhou, Xiaoyong He

**Affiliations:** a Department of Information Science, Zhanjiang Preschool Education College Zhanjiang 524084 Guangdong China; b College of Electronic and Information Engineering, Guangdong Ocean University Zhanjiang 524088 China wangji@gdou.edu.cn; c Guangdong Provincial Smart Ocean Sensing Network and Equipment Engineering Technology Research Center Zhanjiang 524088 China; d School of Electrical Engineering and Intelligentization, Dongguan University of Technology Dongguan 523808 China hxy@dgut.edu.cn

## Abstract

This work employs the femtosecond laser-ablation spark-induced breakdown spectroscopy (fs-LA-SIBS) technique for the quantitative analysis of magnesium alloy samples. It integrates four machine learning models: Random Forest (RF), Support Vector Machine (SVM), Partial Least Squares (PLS), and *k*-Nearest Neighbors (KNN) to evaluate their classification performance in identifying magnesium alloys. In regression tasks, the models aim to predict the content of four elements: manganese (Mn), aluminum (Al), zinc (Zn), and nickel (Ni) in the samples. For classification tasks, the models are trained to recognize different types of magnesium alloy samples. Performance evaluation is based on sensitivity, specificity, and accuracy. The results indicate that the RFR model performs optimally for regression tasks, while the Random Forest Classification (RFC) model outperforms other models in classification tasks. This work confirms the feasibility of quantitative analysis and identification of magnesium alloys using the fs-LA-SIBS technique combined with machine learning methods. It establishes a technical foundation for real-time monitoring of alloys in subsequent laser-induced breakdown spectroscopy (LIBS) instruments.

## Abstract 2

This work employs the femtosecond laser-ablation spark-induced breakdown spectroscopy (fs-LA-SIBS) technique for the quantitative analysis of magnesium alloy samples.

## Graphical abstract

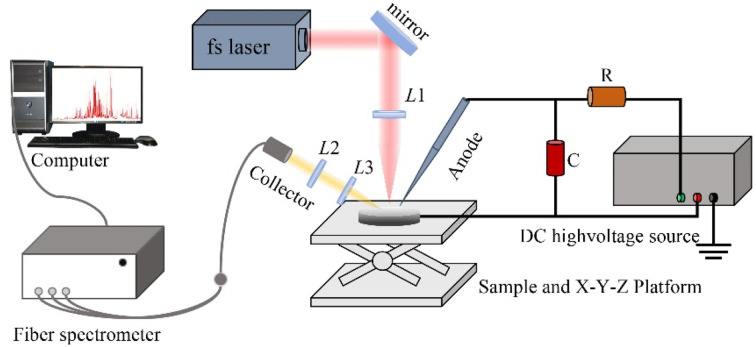

## 1 Induction

With the growing technological demands of society, the application of magnesium alloys in modern industry and daily life is becoming increasingly widespread. Magnesium alloys are materials formed by alloying magnesium with other metals such as manganese (Mn), aluminum (Al), zinc (Zn), nickel (Ni), and rare earth metals, and possibly non-metallic elements. Renowned for their lightweight, high strength, and excellent damping properties, magnesium alloys have found extensive use in fields such as aerospace,^1^ automotive manufacturing,^2^ and consumer electronics.^3^ Therefore, it is very important to use precise analysis techniques to strictly control the elemental content in the production process of magnesium alloys. This method ensures that the chemical composition of the alloys meets strict standards, maintains the stability and consistency of product performance, and also improves production efficiency and the economic benefits of the materials. Femtosecond laser-induced breakdown spectroscopy (fs-LIBS) offers a rapid and minimally destructive method for elemental analysis,^4–6^ ideal for on-site applications without the need for complex sample pretreatment. It enables the simultaneous detection of multiple elements, proving essential across various fields such as materials science, environmental monitoring,^7,8^ industrial quality control,^9,10^ and geological exploration.^11^ The technique requires only a minimal material removal rate, thereby minimizing sample damage and allowing for precise, micro-to-nanometer scale detection. This capability significantly enhances the potential for high spatial resolution in localized elemental analysis.^12^ Moreover, fs-LIBS effectiveness is supported by the unique interaction dynamics between femtosecond lasers and materials, with titanium-sapphire femtosecond lasers providing robust single-pulse energy and high repetition rate capabilities, which further refine its analytical performance.^13,14^

In fs-LIBS analysis, the duration of atomic emission generated by ablating samples with femtosecond lasers is usually shorter than that when using nanosecond lasers. This is a distinct disadvantage in spectral analysis as it directly leads to lower detection sensitivity of elements, especially for alloy element analysis. However, femtosecond lasers offer significant advantages in reducing background noise, improving spatial resolution, and minimizing matrix effects. Employing spark discharge to enhance the optical radiation of the plasma can significantly improve the sensitivity of spectral analysis. For terminological simplicity, this technique is termed femtosecond laser-ablation spark-induced breakdown spectroscopy (fs-LA-SIBS). The spark discharge LIBS (SD-LIBS) system combines high-voltage fast discharge circuits with traditional LIBS experimental setups.^15–18^ Due to the lower energy requirement of laser pulses, this method induces minimal damage to the sample surface. Furthermore, experiments have shown that under discharge conditions, the sample is ablated solely by the femtosecond laser. Therefore, the diameter of the craters on the sample surface is determined primarily by the characteristics of the femtosecond laser, including its focusing properties, pulse energy, and density. The discharge does not affect the crater diameter. This shows the biggest advantage of using femtosecond lasers as ablation sources is that they allow for high spatial resolution elemental analysis of micro-areas within the sample.^19^ These advantages make fs-LA-SIBS tech particularly effective for the quantitative analysis and identification of magnesium alloy samples. The aim of this work is to further optimize the performance of fs-LA-SIBS technique in magnesium alloy analysis by integrating machine learning methods, thereby achieving higher precision and reliability in the results.^19^

Machine learning methods applications in LIBS technique mainly include clustering, classification, and regression.^20^ Clustering, an unsupervised learning technique, forms multiple clusters with distinct centers solely based on the features of the data without the need for prior class labels.^21^ Classification, on the other hand, is a supervised learning method that involves learning patterns from sample data and class labels.^22^ Regression, also supervised, learns the relationship between continuous outcomes, forming patterns between the true values of spectra and sample results to provide predictive results.^23^ In machine learning clustering analysis, Dong *et al.* combined principal component analysis (PCA) with K-means clustering to classify coal. PCA reduces the dimensionality of the input LIBS spectral data to two principal components, aiming to describe data features with fewer variables. The accuracy of the K-means model based on PCA was found to be 92.59%. However, K-means relies heavily on distance calculation and cluster centers, making it challenging to establish complex separation boundaries between data categories due to its requirement for highly separated sample clusters.^24^ Yu *et al.* classified jade samples from five different locations using LIBS spectral data, employing methods such as partial least squares discriminant analysis (PLS-DA), pairwise PLS-DA, linear discriminant analysis (LDA), and support vector machine (SVM) models.^25^ The results indicated that the nature of the model itself, along with the selection of appropriate feature spectral lines based on weight differences, led to superior performance of the SVM model. The high accuracy demonstrated the suitability of LIBS technique for origin classification.

The Random Forest Regression (RFR) model can better uncover patterns in data, filtering out or discarding features with poor correlations, and using highly correlated data to build machine learning models, thereby improving model fitting and robustness. Li *et al.* proposed a new method that combines LIBS and RFR for the quantitative analysis of multiple elements in steel samples.^26^ This method utilizes normalized LIBS spectra to establish a calibration model by optimizing RFR parameters and comparing the performance of different input variables. The study results demonstrate the potential application value of integrating LIBS technique with RFR models for rapid *in situ* determination of multiple elements, particularly in the metallurgical field. Yang *et al.* utilized the RFR-LIBS model to measure the basicity of 30 sintered ore samples.^27^ They optimized the parameters of the RFR model, validated its prediction accuracy through a test set, and found that the RFR model outperformed the PLSR model. This technique shows promise as a method for real-time online rapid analysis in the mining industry. Liu *et al.* investigated the combination of LIBS with variable importance-based RFR (VI-RFR) for the quantitative analysis of toxic elements (Pb, Cr, and Hg) in plastic products.^28^ The results demonstrated that the LIBS-VI-RFR model exhibited superior performance in the quantification of Pb, Hg, and Cr in plastics, with lower root mean square error and higher correlation coefficients compared to other methods. Wang *et al.* proposed an RFR model combining LIBS and infrared spectroscopy (IR) data fusion for identifying different geographical regions of Radix Astragali. LIBS and IR spectra of 19 samples were collected and analyzed.^29^ The results showed that the predictive performance of the RF model based on data fusion surpassed that of individual LIBS or IR methods. Among them, the RF model based on intermediate-level data fusion exhibited the best performance, with high sensitivity, specificity, and accuracy.

This work introduces a rapid identification analysis method for magnesium alloys, based on standard magnesium alloy samples, employing the fs-LA-SIBS technique in combination with machine learning. Recognition models such as RF, SVM, PLS-DA, and KNN were established and compared for their effectiveness in processing fs-LA-SIBS data. Furthermore, the performance of these models was assessed through sensitivity, specificity, and accuracy metrics, discussing their potential applications in metallurgical analysis and identification.

## 2 Experimental

**Fig. 1:**
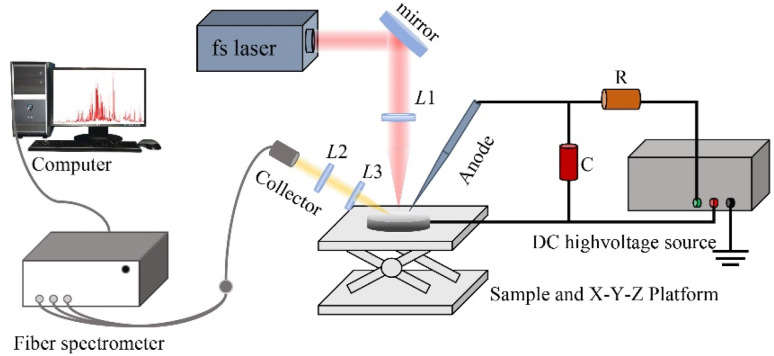
Schematic diagram of fs-LA-SIBS.

The experimental setup for the fs LA-SIBS technique is illustrated in Fig. 1. A Ti:sapphire femtosecond laser system (Coherent Inc., model Astrella-Tunable-USP-1K) operating at a repetition rate of 1 kHz serves as the ablation laser source, with a wavelength, pulse width, and pulse energy of 800 nm, 35 fs, and 7.5 mJ, respectively. The femtosecond laser beam has a diameter of 10 mm. A direct-current high voltage power supply (10 kV, 200 mA) is employed for spark discharge, where the capacitor C is charged through a 100 kΩ limiting resistor R. A tungsten needle with a diameter of 2 mm acts as the anode in the discharge circuit, while a magnesium alloy sample serves as the cathode. The tungsten needle is positioned horizontally at a 45° angle relative to the sample surface, with its tip 2 mm away from the sample surface. The experimental setup operates in a laser-triggered spark discharge mode, when the laser passes through the focusing lens L1 (*f* = 150 mm) to ablate the sample, plasma is generated, triggering the spark discharge circuit immediately to obtain enhanced plasma emission. The enhanced plasma radiation is collected by a quartz lens L2 (*f* = 100 mm) and focused through another quartz lens L3 (*f* = 100 mm) onto the fiber-optic entrance of a compact multi-channel spectrometer (Avantes, AVS-desktop-USB2). The spectrometer has a resolution of 0.15 nm in the wavelength range of 200–500 nm and is equipped with a 2048-pixel charge-coupled device (CCD) operating in a non-gated mode. In this work, the discharge voltage, capacitance, and laser pulse energy are set to 2 kV, 5 nF, and 1.2 mJ, respectively.

The experiment analyzed standard magnesium alloy samples purchased from Aluminum Corporation of China Limited. Table 1 lists the concentrations of different elements in 5 different numbered standard samples. The element contents in the samples were determined using ICP-MS and AAS techniques, which are widely recognized for their accuracy and precision in trace element analysis. These techniques were calibrated and optimized specifically for the sample matrix to ensure reliable and reproducible results. Based on established literature and manufacturer specifications, the uncertainties associated with these measurements typically range from 2% to 10%, depending on the experimental conditions and sample characteristics. Among these 5 samples, particular attention was paid to the concentrations of aluminum (Al), manganese (Mn), nickel (Ni), and zinc (Zn). These spectral data were used for model training and calibration, with 70% randomly selected as the training set and the remaining 30% used as the test set. The training set was used for establishing the multivariate calibration model and optimizing model parameters, while the test set was used to verify the accuracy of the model's quantitative analysis results. Each sample collected 100 sets of fs-LA-SIBS spectral data, with each spectrum averaged 500 times by the fiber optic spectrometer and an integration time set to 10 ms. The spectral data covered a wavelength range from 200 nm to 500 nm, with each data set containing 5958 data points.

**Table 1:** Content of manganese (Mn), aluminum (Al), zinc (Zn), and nickel (Ni) elements in magnesium alloy (wt%)

Sample no.	Concentration (%)			
	Mn	Al	Zn	Ni
1# (G301)	0.082	3.04	1.21	0.0006
2# (G302)	0.256	5.06	0.95	0.0047
3# (G303)	0.374	6.97	0.71	0.0096
4# (G304)	0.57	9.00	0.46	0.015
5# (G305)	0.71	10.4	0.201	0.019

## 3 Methodology

### 3.1 The RF algorithm

**Fig. 2:**
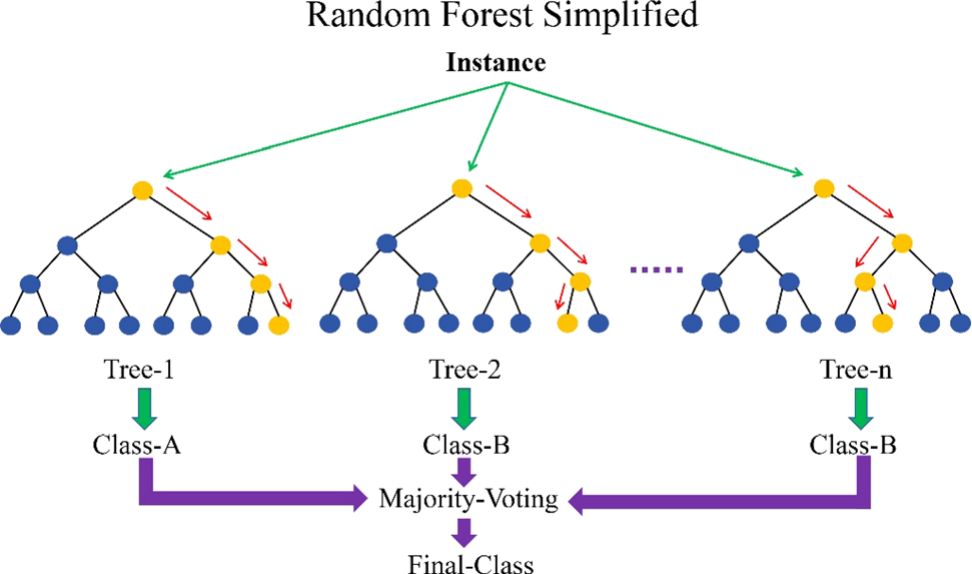
The schematic diagram of the random forest principle.

The random forest regression algorithm falls under the umbrella of ensemble learning techniques, which amalgamate multiple learning algorithms with varying efficiencies to enhance the overall learning efficacy of the model. The schematic diagram of the random forest principle is shown in Fig. 2. Comprising numerous decision trees, the RF model presents as a versatile ensemble method for classification and regression tasks. Each decision tree within the forest operates independently, contributing to the collective wisdom of the model. In essence, a decision tree embodies a tree-like structure where nodes represent feature attributes, and branches delineate the decision-making process based on those attributes. This hierarchical approach facilitates efficient classification, as the sample's category is determined by traversing down the tree from the root node to the leaf nodes, guided by the features at each node. The process of constructing a decision tree model involves several steps. Initially, a subset of features and corresponding labels is randomly sampled from the dataset. Then, at each node, a subset of dimensions from the feature space is chosen to split the data into distinct categories. This partitioning continues recursively until certain stopping criteria are met, such as reaching a maximum tree depth or achieving purity in the leaf nodes. To create a random forest regression, this decision tree construction process is repeated multiple times, with each iteration generating a new tree. By leveraging random sampling and feature selection, each decision tree in the forest is trained on a slightly different subset of data, introducing diversity and reducing overfitting. Through this ensemble approach, the RF model harnesses the collective wisdom of multiple decision trees to deliver robust and accurate predictions. Moreover, the inherent randomness in feature selection and data sampling helps mitigate bias and variance, enhancing the model's generalization capabilities. The RF model algorithm excels in handling complex datasets by leveraging the power of ensemble learning, where the synergy of diverse decision trees yields superior predictive performance and robustness.

### 3.2 Evaluation metrics for random forest regression

Regression is one of the fundamental directions in supervised learning. Computers extract features from the data and labels in the training set, predicting one of the labels for each data point in the test set, with the labels being continuous. In LIBS, regression algorithms are utilized by using the features of different sample spectra as data and the elemental content or other information of the samples as labels for learning. Subsequently, they predict the labels for test sample spectra based on their features. When evaluating the predictive performance of machine learning models, the coefficient of determination (*R*^2^), root mean square error (RMSE), and mean relative error (MRE) are three important metrics in regression models. The formula of three important metrics the coefficient of determination (*R*^2^, RMSE, and MRE) are as follows:



1

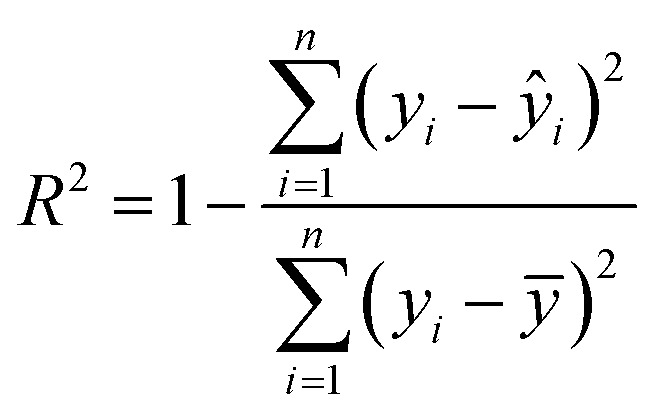



2

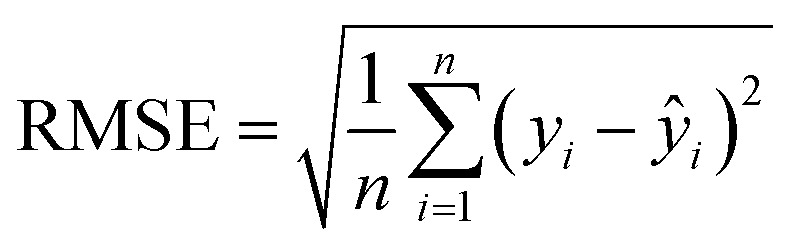



3

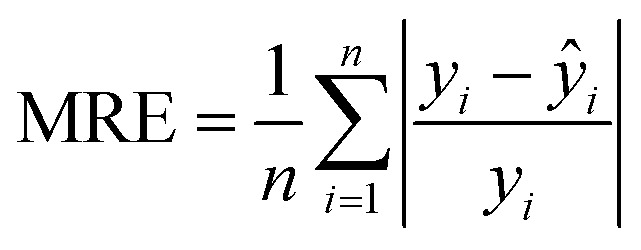

where *n* is the number of samples, *y*_*i*_ is the actual value of the *i*th sample, *ŷ*_*i*_ is the predicted value of the *i*th sample, and *ȳ* is the average of the actual values of the samples.

### 3.3 Evaluation metrics for random forest classification

When evaluating the classification performance of a machine learning model, Sensitivity, Specificity and Accuracy are three important metrics in classification models. The formula of True Positive Rate (TPR), True Negative Rate (TNR), and accuracy are as follows:

4 TPR = TP/(TP + FN)

5 TNR = TN/(TN + FP)

6 Accuracy = (TP + TN)/(TP + TN + FP + FN)

where TP is the number of true positives, FN is the number of false negatives, TN is the number of true negatives and FP is the number of false positives.

## 4 Results and discussion

### 4.1 Enhancement of the fs-LA-SIBS signal

**Fig. 3:**
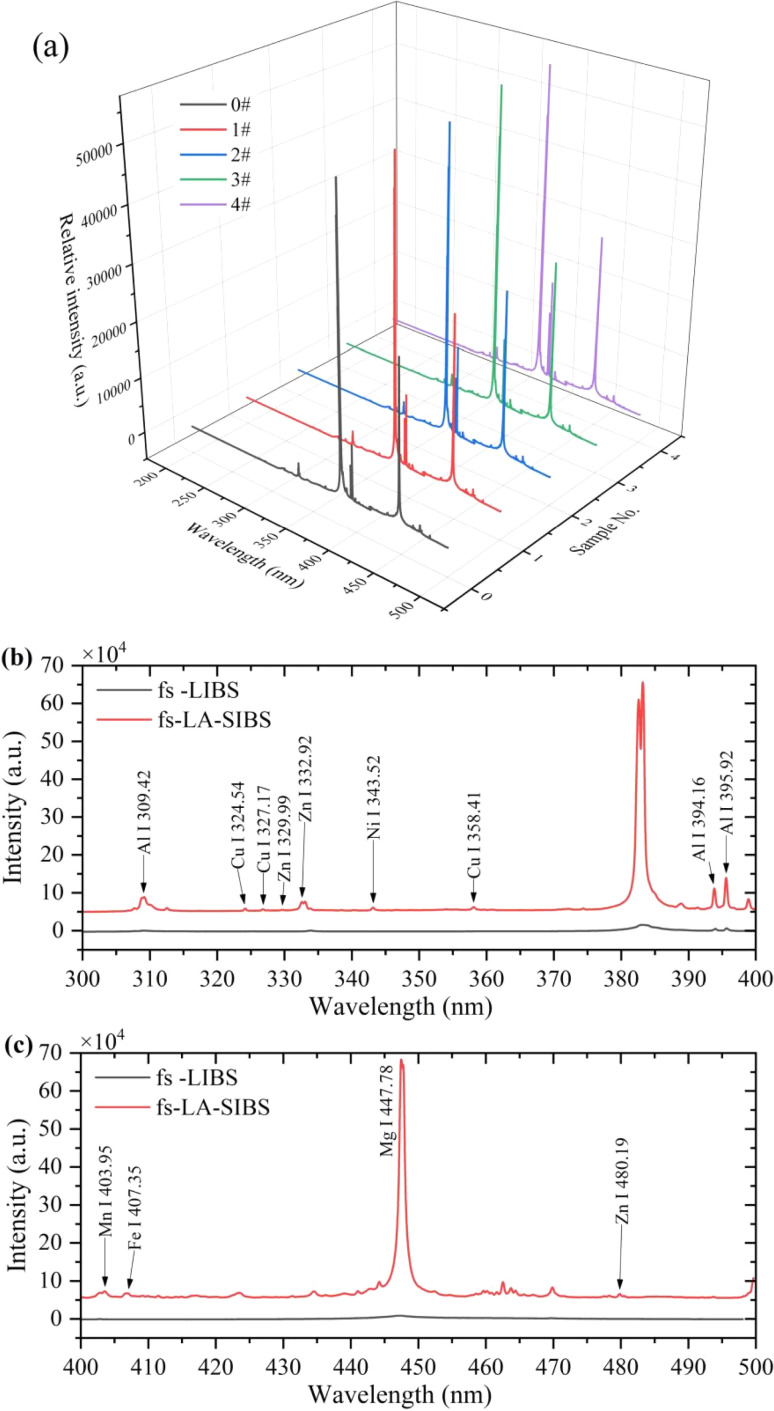
(a) Shows the plasma emission spectra of different magnesium alloy samples at wavelengths from 300 to 500 nm; comparing the plasma spectra recorded in fs-LA-SIBS and fs-LIBS, (b) 300–400 nm and (c) 400–500 nm.

In the fs-LA-SIBS experiment, Fig. 3 shows the spectral comparison of five standard magnesium alloy samples in the wavelength range of 300–500 nm. Due to the high spectral signal intensity of Mg, which exceeds 60 000, only the signals of Al I at 394.40 nm and 396.15 nm can be distinguished, while other trace elements with lower concentrations are difficult to distinguish in the figure. However, the experimental results show that the spectral signals of these elements do exhibit differences. Compared with high-power lasers, using low pulse energy can reduce atomic emission signals and background emissions, which are suitable for none-gated detection methods to capture the emission spectrum. Therefore, this work employed an none-gated optic fiber spectrometer to record the spectrum. The spectrometer did not detect strong signals in the 200–300 nm range, hence Fig. 3(b) and (c) show the plasma emission spectrum observed in the 300–500 nm region using the Avantes multi-channel fiber spectrometer for fs-LIBS and fs-LA-SIBS. The experimental conditions were consistent with those previously described, with an integration time of 10 ms and the spectrum being an average of 500 repeated measurements. The discharge voltage and capacitance were set to 2.0 kV and 10 nF, respectively. Compared to fs-LIBS, the spectrum of fs-LA-SIBS was significantly enhanced, with spark discharge increasing the peak intensity by more than ten-folds.

### 4.2 Selection of input variables

Through the RFR model, performance comparison and analysis were conducted on multi-elements (Mn, Al, Zn, Ni) within different wavelength ranges to explore the influence of wavelength selection on the model predictive accuracy. Table 2 presents detailed performance indicators across two wavelength ranges, 200–500 nm and 280–500 nm.

**Table 2:** The performance of RFR model with different wavelength inputs

Occluded fs-LA-SIBS bands (nm)	Element	*R* ^2^	RMSE	MRE
200–500	Mn	0.978	0.021	0.022
	Al	0.775	10.023	21.081
	Zn	0.663	1.354	0.044
	Ni	0.976	0.676	1.629
280–500	Mn	0.982	0.014	0.018
	Al	0.806	9.862	20.597
	Zn	0.692	1.219	0.035
	Ni	0.981	0.586	1.572

The analysis revealed that by reducing the wavelength range from 200–500 nm to 280–500 nm, the *R*^2^ values for all elements improved, indicating enhanced model prediction capability. Particularly for Mn and Ni, within the 280–500 nm wavelength range, the model demonstrated exceptionally high predictive accuracy, with *R*^2^ values approaching 1. This suggests that the shorter wavelength range better captures the characteristic information of these elements, thereby improving model accuracy.

Compared to Mn and Ni, the performance of the model for Al showed poorer performance in both wavelength ranges. Although there was an improvement in predictive accuracy after reducing the wavelength range, RMSE and MRE remained relatively high. While the predictive performance of Zn improved after reducing the wavelength range, there still exists a significant gap compared to Mn and Ni.

Although the numerical improvement is small, in the context of complex magnesium alloy spectral data, it shows that the selection of wavelength ranges influences the optimization of the performance of RFR model in predicting metal element content. These small improvements could have a significant impact in real-world applications, and greater performance gains will be realised in the future by incorporating additional techniques.

### 4.3 Optimization of the RFR model through Out-of-Bag (OOB) error

To optimize the performance of the RFR model, this work employed the Out-of-Bag (OOB) error method to compare the effects of different numbers of decision trees (*n*_tree_) and feature trees (*m*_try_). *N*_tree_ represents the number of trees in the forest, where increasing the number of trees typically improves the model performance until a certain point, beyond which the improvement becomes marginal. However, more trees also entail longer training times and higher computational costs. *M*_try_ denotes the number of features randomly selected at each node when splitting *n*_tree_. This random selection of a subset of features for each split increases model diversity, reduces overfitting risks, and enhances the model generalization ability.

**Fig. 4:**
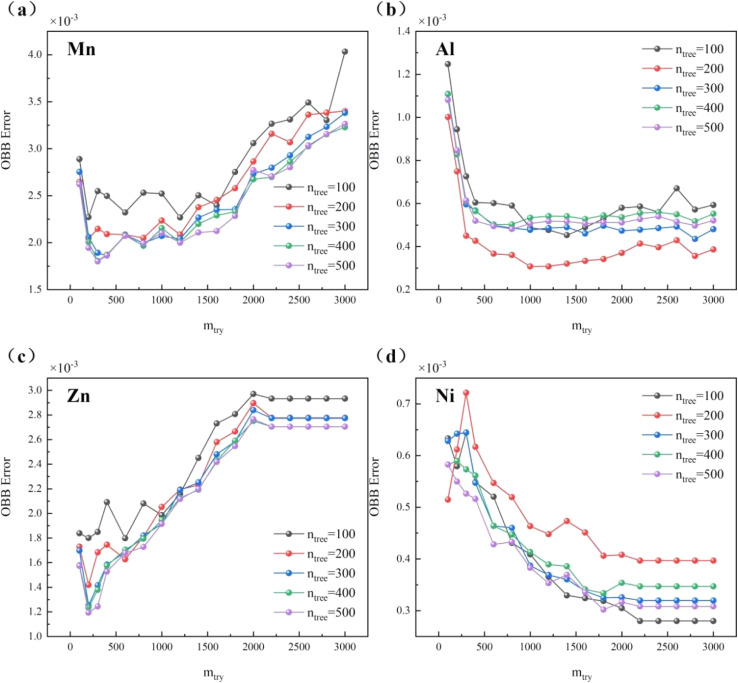
The influence of different parameters (*n*_tree_ and *m*_try_) on OOB Error in RFR model.

Optimizing the number of *n*_tree_ and feature selection not only enhances the predictive performance of the RFR model but also improves its efficiency and applicability. As depicted in Fig. 4(a), the regression model for manganese achieved excellent performance at *n*_tree_ = 500, *m*_try_ = 300, achieving high performance at a relatively low computational cost. In Fig. 4(b), the regression model for aluminum performed well at *n*_tree_ = 200, *m*_try_ = 1000, demonstrating high performance with limited computational resources. In Fig. 4(c) and (d), the optimal configurations for the regression models of zinc and nickel were *n*_tree_ = 500, *m*_try_ = 200, and *n*_tree_ = 300, *m*_try_ = 2200, respectively, confirming that high predictive accuracy can be achieved under limited computational resources.

Table 3 presents the predictive performance of the RFR model for specific elements (Mn, Al, Zn, Ni) before and after optimization, including *R*^2^, RMSE, and MRE. Before optimization, the model already exhibited high predictive accuracy for Mn, with an *R*^2^ value of 0.977616. The predictive accuracy for Al was relatively low, with an *R*^2^ of only 0.774895. Zn showed high predictive performance similar to Mn, while Ni had the lowest predictive performance, with an *R*^2^ value of only 0.662897. After optimization, the predictive performance of all elements improved. The *R*^2^ value for Mn increased to 0.994041, with significantly reduced RMSE and MRE, indicating a significant improvement in Mn element predictive accuracy. The *R*^2^ of Al also increased, with slight reductions in RMSE and MRE compared to Mn. After optimization, Zn exhibited the best predictive performance, with an *R*^2^ value as high as 0.998904 and RMSE and MRE close to 0, indicating nearly perfect prediction. The performance of Ni also improved after optimization, with improvements in *R*^2^, RMSE, and MRE.

**Table 3:** The performance of RFR model before and after OOB error optimization

OOB error optimization	Element	*R* ^2^	RMSE	MRE
Before optimization	Mn	0.978	0.021	0.022
	Al	0.775	10.023	21.081
	Zn	0.976	1.354	0.044
	Ni	0.663	0.676	1.629
After optimization	Mn	0.994	0.008	0.009
	Al	0.837	8.269	18.200
	Zn	0.999	0.005	0.010
	Ni	0.740	0.470	0.976

These results demonstrate the significant positive impact of OOB error optimization on the performance of the RFR model, particularly in reducing prediction errors. This renders the model more apt for predicting intricate datasets, particularly when handling elements with varying degrees of variability.

### 4.4 Evaluation of RFC model classification performance

**Fig. 5:**
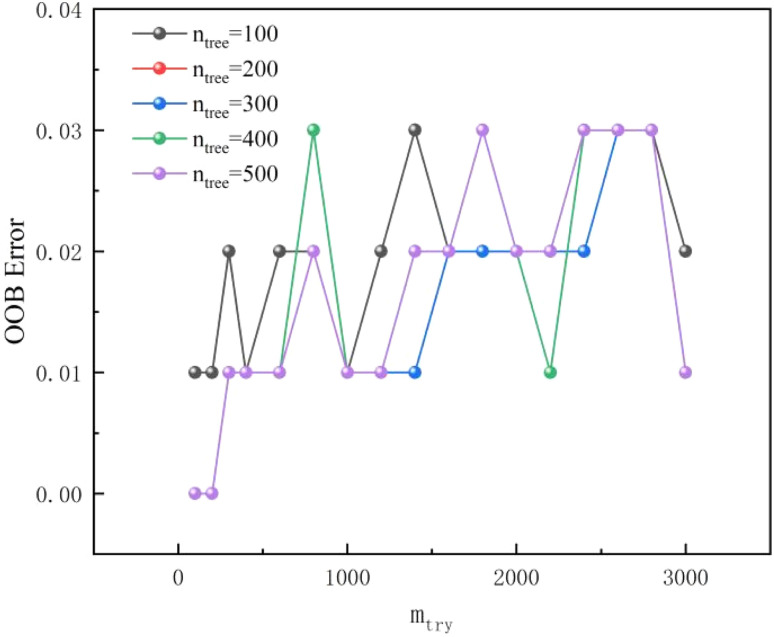
The influence of parameters *n*_tree_ and *m*_try_ in RFC model on OOB error.

By employing the OOB error method, the classification accuracy of the random forest classification model has been enhanced, along with further improvements in its operational efficiency and applicability. As depicted in Fig. 5, the optimal parameters are achieved when *n*_tree_ is set to 100 and *m*_try_ is also set to 100, resulting in a model error rate of 0. However, in typical scenarios, a non-zero error rate is more in line with practical expectations, as achieving perfect prediction accuracy is unlikely in most cases. Therefore, to ensure data accuracy, as a precautionary measure, parameters of *n*_tree_ = 100 and *m*_try_ = 100 are chosen.

Table 4 displays the classification performance of the RFC model on different samples before and after optimization. Before optimization, both sensitivity and specificity for sample no. 1# were 1.000, indicating the model perfect prediction accuracy for this sample. However, the accuracy slightly fell below 1 (0.975) due to misclassifications in other sample categories. For sample no. 2#, sensitivity and specificity before optimization were 0.942, with an accuracy of 0.937, indicating good performance in identifying this sample but with room for improvement. Sample no. 3# exhibited lower sensitivity (0.578) before optimization, despite higher specificity (0.933), yet achieving an accuracy of 1, indicating high overall classification accuracy despite insufficient positive identification capability. This may suggest imbalanced class distribution in the classification problem or complete and accurate classification of samples in other categories. Sensitivity and specificity for sample no. 4# and 5# demonstrated excellent performance before optimization, with high accuracy, particularly achieving perfect accuracy for sample no. 5#. After optimization, sensitivity and specificity for all samples reached 1.000, indicating perfect performance in identifying positive and negative instances. Except for sample no. 4# with an accuracy of 0.754, all other samples achieved perfect accuracy of 1.000. The parameters adjusted during the optimisation process may not have a significant effect on sample no. 3#, but have a significant effect on other samples, resulting in the model not improving its accuracy relative to other samples in recognising that class of samples very significantly. Or perhaps the diversity of sample no. 3# in the training set is insufficient to cover all variants of the samples in this class, resulting in insufficient generalisation ability of the model.

**Table 4:** Evaluation of RFC model classification performance before and after OOB error optimization

OOB error optimization	Sample no.	Sensitivity	Specificity	Accuracy
Before optimization	1#	1.000	1.000	0.975
	2#	0.942	0.942	0.937
	3#	0.578	0.933	1.000
	4#	1.000	0.917	0.722
	5#	0.964	0.964	1.000
After optimization	1#	1.000	1.000	1.000
	2#	1.000	1.000	1.000
	3#	0.667	1.000	1.000
	4#	1.000	0.941	0.754
	5#	1.000	1.000	1.000

Overall, the optimized RFC model exhibited significant performance improvements across most samples.

**Fig. 6:**
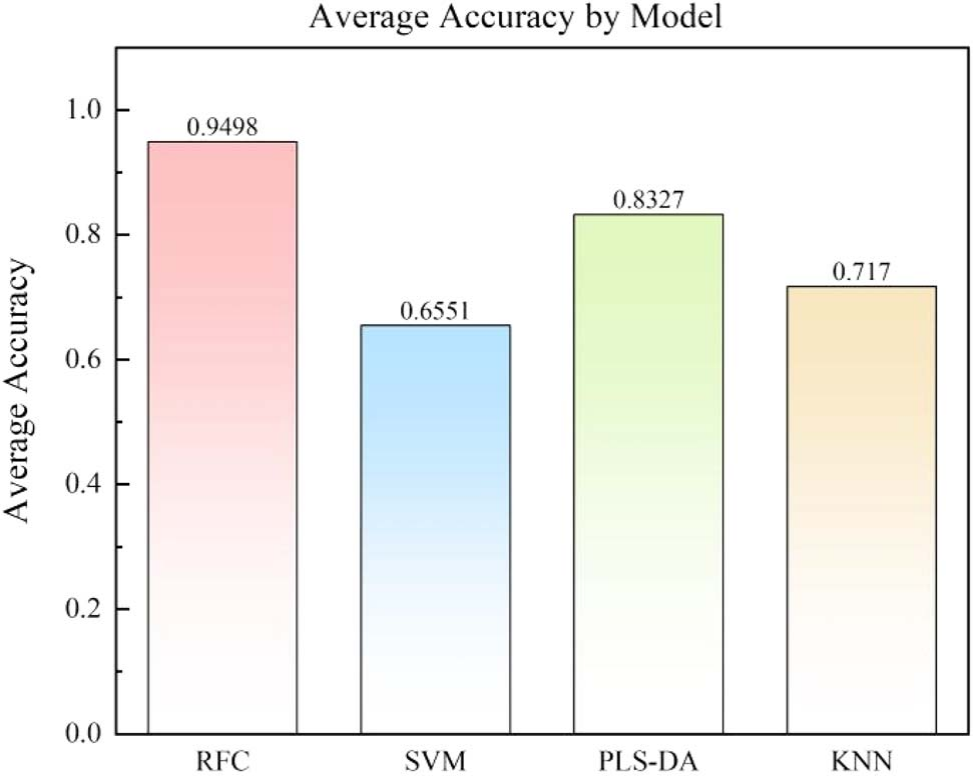
Average accuracy after 100 independent classifications of different models.

Fig. 6 presents the average accuracies of the four models after 100 independent tests. The results demonstrate that the RFC model achieves the highest average accuracy of 0.9498, confirming its outstanding performance in this task. In comparison, the SVM model has an average accuracy of 0.6551, PLS-DA model has 0.8327, and KNN model has 0.7170, all significantly lower than the RFC model. Therefore, the RFC model indisputably emerges as the preferred choice for magnesium alloy classification tasks. This finding not only provides valuable insights for alloy classification but also sets a precedent for the application of machine learning in materials science. The work further underscores the practicality and reliability of the RFC model, offering strong support for future industrial production and materials research endeavors.

## 5 Conclusion

This work utilizes fs-LA-SIBS combined with machine learning algorithms to analyse the elemental content in magnesium alloy samples as well as model identification. The RFR model demonstrated excellent predictive accuracy with a high *R*^2^ (no less than 0.740), low RMSE (no greater than 8.269) and MRE (no greater than 18.200). In the classification task, the RFC model is able to show better performance in dealing with unbalanced datasets with higher evaluation capabilities (sensitivity not less than 0.667, specificity not less than 0.941, and accuracy not less than 0.754). This work not only introduces novel technical means for the rapid and accurate classification of alloy samples but also lays a robust theoretical foundation and practical solutions for material identification in related industrial domains. Future endeavors could delve deeper into exploring the RFR model application in broader material classification tasks and optimizing its parameters to accommodate more complex real-world scenarios.

## Data availability

The data that support the findings of this study are available upon reasonable request from the author.

## Author contributions

Jun Liu: writing – original draft, methodology, funding acquisition, formal analysis, and supervision. Ji Wang: writing – review & editing, methodology, data curation, formal analysis, software, funding acquisition, and conceptualization. Xiaopei Li: writing – original draft, conceptualization, data curation, and formal analysis. Hai Lin: methodology, investigation, formal analysis, and software. Tiancheng Liu: writing – original draft, methodology, and formal analysis. Bingyan Zhou: methodology, data curation, and formal analysis. Xiaoyong He: writing – review & editing, methodology, conceptualization, and supervision.

## Conflicts of interest

The authors declare that they have no known competing financial interests or personal relationships that could have appeared to influence the work reported in this paper.
